# Energy Expenditure as a Function of Activity Level After Spinal Cord Injury: The Need for Tetraplegia-Specific Energy Balance Guidelines

**DOI:** 10.3389/fphys.2018.01286

**Published:** 2018-09-19

**Authors:** Jessie R. Shea, Barbara L. Shay, Jeff Leiter, Kristine C. Cowley

**Affiliations:** ^1^Department of Physiology and Pathophysiology, Rady Faculty of Health Sciences, University of Manitoba, Winnipeg, MB, Canada; ^2^Department of Physical Therapy, College of Rehabilitation Sciences, Rady Faculty of Health Sciences, University of Manitoba, Winnipeg, MB, Canada; ^3^Department of Surgery, Rady Faculty of Health Sciences, University of Manitoba, Winnipeg, MB, Canada; ^4^Pan Am Clinic Foundation, Winnipeg, MB, Canada

**Keywords:** dietary guidelines, obesity, paraplegia, sedentary-related diseases, body composition, type II diabetes, resting energy expenditure, exercise-based energy expenditure

## Abstract

The World Health Organization recognizes obesity as a global and increasing problem for the general population. Because of their reduced physical functioning, people with spinal cord injury (SCI) face additional challenges for maintaining an appropriate whole body energy balance, and the majority with SCI are overweight or obese. SCI also reduces exercise capacity, particularly in those with higher-level injury (tetraplegia). Tetraplegia-specific caloric energy expenditure (EE) data is scarce. Therefore, we measured resting and exercise-based energy expenditure in participants with tetraplegia and explored the accuracy of general population-based energy use predictors. Body composition and resting energy expenditure (REE) were measured in 25 adults with tetraplegia (C4/5 to C8) and in a sex-age-height matched group. Oxygen uptake, carbon dioxide production, heart rate, perceived exertion, and exercise intensity were also measured in 125 steady state exercise trials. Those with motor-complete tetraplegia, but not controls, had measured REE lower than predicted (mean = 22% less, *p* < 0.0001). REE was also lower than controls when expressed per kilogram of lean mass. Nine had REE below 1200 kcal/day. We developed a graphic compendium of steady state EE during arm ergometry, wheeling, and hand-cycling. This compendium is in a format that can be used by persons with tetraplegia for exercise prescription (calories, at known absolute intensities). EE was low (55–450 kcal/h) at the intensities participants with tetraplegia were capable of maintaining. If people with tetraplegia followed SCI-specific activity guidelines (220 min/week) at the median intensities we measured, they would expend 563–1031 kcal/week. Participants with tetraplegia would therefore require significant time (4 to over 20 h) to meet a weekly 2000 kcal exercise target. We estimated total daily EE for a range of activity levels in tetraplegia and compared them to predicted values for the general population. Our analysis indicated that the EE values for sedentary through moderate levels of activity in tetraplegia fall well below predicted sedentary levels of activity for the general population. These findings help explain sub-optimal responses to exercise interventions after tetraplegia, and support the need to develop tetraplegia-specific energy-balance guidelines that reflects their unique EE situation.

## Introduction

For everyone, energy used by the body must at least equal energy intake or body fat will increase, which eventually may result in obesity and increased risk of related diseases.^1^ The World Health Organization indicates that today, the world is facing a global epidemic of overweight and obesity related malnutrition.^2^ Individuals with spinal cord injury (SCI), and especially those with cervical SCI (tetraplegia), rank at the lowest end of the fitness continuum (LaPorte et al., 1984), and have a higher incidence of obesity (Spungen et al., 2003), type II diabetes and impaired glucose tolerance (Duckworth et al., 1980; Bauman and Spungen, 1994), dyslipidemia (Bauman et al., 1992) and cardiovascular disease risk (Gibson et al., 2008) compared to the general population. It is clear that balancing macro-nutrient or energy intake (calories) to energy expenditure is critical for maintaining a healthy body composition and reducing the risk of developing sedentary diseases. Although it is recognized that people with SCI should consume fewer calories than recommended for the general population, dietary energy consumption guidelines for the SCI population as a whole do not exist.

Spinal cord injury reduces resting energy expenditure (REE) compared to the general population (Sedlock and Laventure, 1990; Monroe et al., 1998; Buchholz et al., 2003; Jeon et al., 2003). When expressed per kilogram of lean tissue, some report REE remains lower in SCI participants compared to controls (Sedlock and Laventure, 1990; Monroe et al., 1998) whereas others report the differences became non-significant (Buchholz et al., 2003; Jeon et al., 2003). In addition, discrepancies exist as to whether those with tetraplegia have lower REE than those with paraplegia. One research study reported tetraplegia-level injury had lower REE per kilogram body weight (Mollinger et al., 1985), whereas two others reported similar REE (Collins et al., 2010; Holmlund et al., 2018), but none of these studies measured body composition. Therefore, we measured resting energy use and DXA-based body composition in a group of male and female participants with tetraplegia.

Exercise guidelines for the general population are well established, and recommend approximately 150 min of moderate to vigorous exercise per week to reduce risk of cardiovascular and other sedentary-related diseases such as obesity [e.g.,^3^ or (Who, 2010; Tremblay et al., 2011)]. In terms of cardiovascular disease and the volume of exercise needed to reduce disease risk after SCI, the recommendation that “exercise guidelines for people with SCI should be consistent with those for the general population” (Kressler et al., 2014; Tweedy et al., 2016) has been generally supported by systematic review of the SCI exercise research literature (Martin Ginis et al., 2017). However, SCI-specific guidelines are based mainly on studies involving people with paraplegia (van der Scheer et al., 2017). Tetraplegia-specific research is needed because although tetraplegia typically comprises between 40 and 60 percent of the population with traumatic SCI (Noonan et al., 2012; Lee et al., 2014; Singh et al., 2014), systematic reviews of exercise intervention research indicate there is insufficient tetraplegia-specific data to make exercise prescriptions based on injury level (Bochkezanian et al., 2015; van der Scheer et al., 2017). Given the additional functional limitations and impaired sympathetic nervous system function during exercise in tetraplegia (Haisma et al., 2006; Cowley, 2018), it raises the question of whether 150 min weekly of exercise at moderate to high intensity would provide a similar benefit for those with tetraplegia compared to lower level SCI (paraplegia). Therefore it is of interest to assess steady state energy consumption over the range of intensities and modes of exercise possible after tetraplegia, and to provide this information in a format useful to those living with tetraplegia who want to know how many calories they are using, and at known absolute intensities of exercise.

Finally, given the absence of energy intake (calories) guidelines for those with SCI, and the increased physical limitations of tetraplegia, it is of interest to estimate hypothetical total daily energy expenditure (TDEE) values and compare these to caloric intake recommendations for the general population. Such a comparison would be a necessary first step in assessing whether there is a need to develop energy balance guidelines that better reflect the situation experienced by those living with tetraplegia. TDEE based on measured values over a reasonable range of activity levels could be used to help maintain or achieve a healthy percent body fat. Preliminary results have appeared in abstract form (Shea et al., 2013).

## Materials and Methods

### Participant Recruitment

Adults with tetraplegia (*n* = 25) were recruited through the Manitoba Wheelchair Sport Rugby Team and the Manitoba Division of the Canadian Paraplegic Association, flyers posted at the Provincial SCI outpatient clinic and word of mouth. Able-bodied, age-, sex-, and height-matched controls (*n* = 23) were recruited by word of mouth. Study procedures used were reviewed and approved by the University of Manitoba Health Research Ethics Board, and all study participants provided written informed consent in accordance with the Declaration of Helsinki. In addition, all study participants provided written informed consent allowing the release and publication of the demographic information presented in **Table 1**. Energy expenditure measurements took place over two to four study visits for participants with tetraplegia, depending on the number of measurements taken, and over two visits in the able-bodied cohort.

**Table 1:** Participant characteristics demonstrate the range of injury levels and durations, ages, and levels of function in the study sample (*n* = 25).

ID#	Age (years)	Duration of injury (years)	Neurological level of injury	Motor C/I	Ambulation mode	M/F
1	45	25	C8	MC	MWC	F
2	39	19	C6/7	MC	MWC	M
3	55	32	C6/7	MC	MWC	M
4	37	21	C5	MC	PW C	F
5	48	17	LC5/6 RC5/6/7	MC	PW C	M
6	28	12	C6	MC	MWC	M
7	33	1	C5/6	MC	MWC/PWC	M
8	50	31	C6	MC	MWC	M
9	53	16	C6	MC	MWC	F
10	47	17	C4/5	MC	PW C	M
11	44	25	C6/7	MC	PW C	M
12	55	28	C7	MC	MWC	F
13	53	14	C6/7	MC	PW C	M
14	49	1	C5	MC	PW C	F
15	52	25	C8	MI	WFT, no aid, or orthotic	M
16	42	21	LC7 R C6	MC	MWC	M
17	24	4	C5	MI	MWC, WB one leg	M
18	42	23	C6/7	MC	MWC	M
19	48	5	C8	MI	WFT, cane	M
20	70	15	C5/6	MI	WFT, no aid, or orthotic	M
21	40	21	C6	MC	MWC	M
22	57	43	C8	MC	MWC	F
23	38	20	C5/6	MC	MWC	M
24	36	16	C5	MI	W in home, MWC	M
25	22	2	C7/8	MI	MWC	M
Mean Range	44.3 22 - 70	18.2 1 - 43	C4/5 – C8	18 MC:6 MI		6F:18M


*Mean and range are shown at the bottom, where appropriate. L, left; R, right; C, cervical level; MI, motor incomplete; MC, motor complete; WFT, walks full time; WB, weight-bearing; MWC, manual wheelchair; PWC, power wheelchair; F, female; M, male.*

### Anthropometric Measures

Participants were weighed using a digital wheelchair scale (Health O Meter model 2650KL, measures to 0.1 kg) or a Hoyer lift scale for those unable to transfer. Waist circumference was measured at the umbilicus or naval at the end of a normal breath out and hip girth was measured at the widest point, corresponding to level of the greater trochanter. Body length was measured while on a plinth after each person’s body alignment was straightened. A right angle measure was used to line up and mark the position of the top of the head and the sole of the foot. Most participants were measured in one length, although 5 were measured in body segments, due to leg contractures. One person’s contracture precluded measurement and his pre-injury height was used. For age-, sex-, and height-matched controls, height and waist girth were measured while standing and while laying on a plinth in a subgroup (*n* = 20). The average percent difference and standard deviation between measurements taken while supine versus standing in this subgroup were larger for the abdomen than for height (height 0.66 ± 0.74%; waist 2.35 ± 1.77%). Because these values were not significantly different, standing values were used for analysis.

### Body Composition Assessment

An Encore 2500 GE Lunar Prodigy dual energy x-ray absorptiometry (DXA) system was used to assess body composition. Estimates of regional and total body bone mineral content (g), lean and fat tissue mass (kg), and percent of total (%) were calculated. The same trained technician performed all scans and the machine was calibrated daily according to manufacturer’s specifications.

### Energy Expenditure Measurement and Estimation of Energy Use

Oxygen uptake and carbon dioxide production were measured using the Jaeger Oxycon Mobile (Carefusion Respiratory Care, Yorba Linda, CA, United States) exercise testing system. Gas analysers were calibrated and the system thermostated and compensated for barometric pressure and environmental humidity variations before each test, according to the manufacturer’s recommendations (Jaeger Oxycon User Manual). Energy consumption was calculated according to the equations of de Weir (1954). Heart rate was monitored continuously with a chest strap or ear clip-based sensor, based on user preference. Each person rested with the equipment for at least 3 min before testing.

### Resting Energy Expenditure

Following an overnight fast, participants reported to the lab between 08:00 and 11:00. Arrival times later than 09:30 were required for some participants in order to accommodate their attendant-care and transportation-related needs. Measurements were taken for 30 min in a supine position, and data recorded at 5-s intervals were averaged over 5-min intervals. Participants were observed throughout the measurement to ensure they did not fall asleep or talk. Resting energy values were considered steady state when the coefficient of variation (%CV) between values was 5% or less. Values were then averaged over the longest series of consecutive steady-state epochs to determine resting energy consumption.

### Energy Measurement During Exercise

Activities were undertaken for a minimum of 3 min (after attaining the target intensity). Measurements were taken throughout the activity, and data recorded at 5-s intervals were averaged over 30 s epochs. Activity energy values were considered steady-state when the %CV for the energy expenditure values between consecutive 30 s epochs was less than or equal to 10%, similar to methods used previously in the SCI population (Collins et al., 2010). Data were averaged over the longest consecutive series of steady-state epochs.

Steady state exercises were performed based on real-life scenarios and participant capabilities and personal mobility equipment. Arm crank ergometry was performed on a calibrated SCIFit height adjustable arm ergometer, or a table mounted calibrated Colorado arm crank ergometer. Manual wheeling was performed by each person in their own wheelchair on a large level indoor cement track, and hand-cycling with each person’s hand-cycle outdoors on a 400 m track or on the University Campus streets. A bicycle odometer transmitter was placed on each participant’s wheel for real-time monitoring of speed to maintain a steady speed during each trial. The actual wheel rotations were documented using a G-Link accelerometer mounted on a rear wheel axis. Measurement of each person’s wheel circumference allowed conversion of rotations to distance and speed. Accelerometer data were downloaded and files converted for subsequent analysis of rates using custom developed in-house Spinal Cord Research Centre software and Microsoft Excel (2010).

Participants rated subjective exertion on the Borg 1–10 ratings of perceived exertion (RPE) scale at the end of each trial (Borg, 1982). Participants were monitored for symptoms of autonomic dysreflexia, and the exercise protocol was stopped until the symptoms subsided. This occurred in three participants.

### Estimation of Total Daily Energy Expenditure and Comparison to Predicted Values

Estimations were based on an assumption of each person sleeping for 8 h per day combined with increasing durations and/or intensities of daily activity ranging from sedentary (S) to low-to-moderately active (MA) and finally to active (A) using the definitions in Health Canada Guidelines (Government of Canada, 2011). The time spent in each activity is listed in parentheses in **Figure 6** under each activity category title. Predicted, able-bodied values were based on Harris–Benedict resting energy use, and multipliers of resting energy (REE). TDEE values were estimated using the resting and exercise-based data of our male motor-complete sub-group. These values were supplemented with non-exercise activity values estimated using the appropriate multiple of REE taken from either Collins et al. (2010), or Ainsworth et al. (1993), as follows: sleep (0.9 REE), sitting quietly (1.0 REE), walking slowly (2.0 REE), office work (1.7 REE for able-bodied predicted), housework (2.2 REE for able-bodied predicted), moderate exercise (4.5 REE for able-bodied predicted), and high intensity exercise (7.5 REE for able-bodied predicted) from Ainsworth et al. (1993); and deskwork (1.47 REE) and housework (2.1 REE) done by tetraplegia-level injury from Collins et al. (2010). The multiplier of 1.6 REE for indoor wheeling at a low speed, and exercise at moderate (2.4 REE) and high intensity (3.5 REE), are the average multipliers of REE for these activities taken from this report.

### Statistical Analysis

All statistical analyses were performed using GraphPad Prism statistical software (versions 6 & 7; GraphPad Software, La Jolla, CA, United States). The D’Agostino and Pearson omnibus test was used to determine whether data were normally distributed prior to correlation or group analyses and to test residuals after performing linear regression analyses. Depending on the results from normality testing and the relationship being tested, results are reported as either *r*-values for parametric Pearson correlations or as ρ-values for non-parametric Spearman correlations. The co-efficient of determination (r-squared, *R*^2^) is reported for linear regression analyses; residuals were normally distributed unless otherwise noted. *P*-values are indicated for each correlation or linear regression correlation. Two-tailed paired *t*-tests were used to compare two groups of normally distributed data and Sidak’s multiple comparisons test during one-way ANOVA for testing between multiple groups. Non-parametric groups were compared using Dunn’s multiple Mann–Whitney test. For multiple comparisons, the adjusted *p*-value is reported.

## Results

### Participant Characteristics and Study Participation

Participants ranged from 22 to 70 years of age (mean = 44 years), with a mean injury duration of 18 years (range 1–43). Injury levels ranged from C4/C5 to C8, with 21 requiring full-time use of a wheelchair (14 manual, 6 power, 1 person used both). Nineteen of the 25 participants were motor complete (13 men and 6 women), and the remaining six male participants were motor incomplete, with 4 of these participants able to walk full time or while in the home (**Table 1**).

Overall, 25 participants with tetraplegia completed the REE, DXA scan of body composition, and anthropometric measurements (hip and waist girth and height). Data from 22 persons with tetraplegia were suitable for use in resting energy calculations. The reference group consisted of 23 sex, age, and height-matched able-bodied persons from the general population [Age: 23–56, mean = 43; Weight: 54–131 kg, mean = 86 kg; Height: 162–197 cm, mean = 178 cm]. Although the control reference participants were not selected to match for weight, statistical comparison of height, age and weight indicated no significant differences between participants with tetraplegia and our reference group (*n* = 23, paired *t*-test, all *p* > 0.05). As expected, the subgroup of males with motor complete tetraplegia (*n* = 12) had significantly different mean weight (81 kg) compared to their matched reference group (96 kg; paired *t*-test *p* = 0.01).

### Resting Energy Expenditure

**FIGURE 1:**
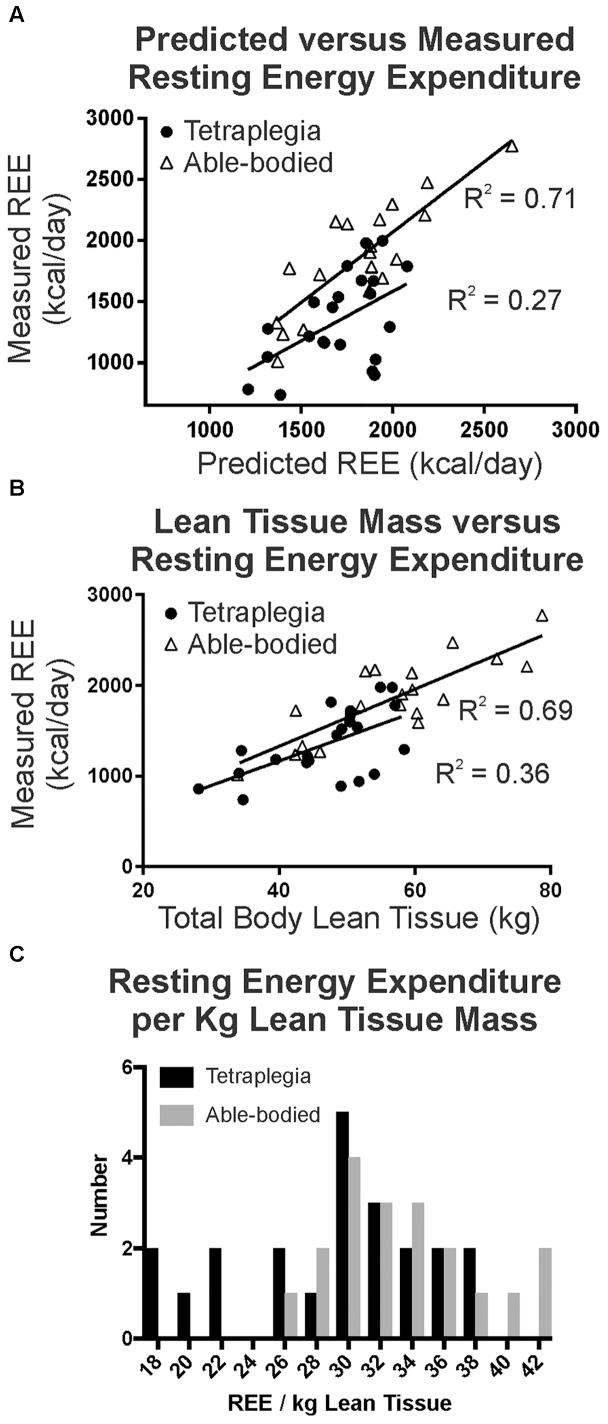
**(A)** A strong correlation between predicted and measured resting energy use is seen in age-height-sex matched controls (*R*^2^ = 0.71), whereas a much weaker correlation existed in participants with tetraplegia (*R*^2^ = 0.27, Pearson, *p* < 0.05). **(B)** Total lean tissue mass better predicted measured REE in able-bodied controls than in participants with tetraplegia. **(C)** Histogram demonstrates a subgroup with tetraplegia had very low REE per kg of lean tissue.

Predictive energy use equations (Harris and Benedict, 1918) were well correlated with measured resting energy expenditure (REE, **Figure 1A**) in our able-bodied control participants (triangles, *R*^2^ = 0.71), and were consistent or better correlated than other general population-based comparisons between measured and predicted REE (Roza and Shizgal, 1984; Bogardus et al., 1986). In contrast, only 27% of the variation was shared between predicted and measured REE (filled circles, *R*^2^ = 0.27) for participants with tetraplegia. Consistent with this observation, resting energy use was significantly lower than predicted in the group with tetraplegia (*p* < 0.0001, paired two-tailed *t*-test) but not in the able-bodied controls (*p* = 0.3325). In addition, measured resting energy use in participants with tetraplegia was significantly less than the measured resting values in matched controls (*p* < 0. 0001). The average measured daily REE was 1414 kcal/day in males with motor complete SCI, which was 21% (386 kcal/day) less than predicted (1800 kcal/day). Similarly, the average measured daily REE was 1104 kcal/day or 22% (309 kcal/day) less than predicted (1413 kcal/day) for the female participants with motor complete SCI. Participants with motor incomplete injury capable of walking full time had a smaller difference between average measured and predicted REE, at 159 kcal/day (9% less), with a measured value of 1672 ± 288 compared to a predicted 1832 ± 142 kcal/day.

There was a positive and very strong correlation between REE and total lean body mass in the able-bodied and a weaker, albeit statistically significant correlation for those with SCI (Evans, 1996). Linear regression indicated that 70% of the variation was shared between total lean tissue mass and measured resting energy use in able-bodied participants (**Figure 1B** triangles, *R*^2^ = 0.69, *r* = 0.83, *p* < 0.0001), but only 36% was shared with tetraplegia (**Figure 1B** filled circles, *R*^2^ = 0.36, *r* = 0.60, *p* = 0.003). Further, visual inspection of **Figure 1B** suggests there may be two distinct groups within the participants with tetraplegia: those that exhibit similar resting energy use to able-bodied controls when total body lean tissue is taken into account, and those that still demonstrate lower resting energy. To test this, we plotted and compared the distribution of measured total daily REE per unit of lean tissue (**Figure 1C**, REE/kg lean tissue) for the SCI (black) and able-bodied (gray) groups and found the difference to be statistically significant (paired two-tailed *t*-test, *p* = 0.0138). Although normalizing REE to lean tissue mass did not account for the difference in REE between the two groups, visual inspection of **Figure 1C** reveals an overlap in the distribution such that a portion of those with tetraplegia show similar REE/kg lean tissue (those with REE > 26 kcal/kg) and a subgroup with much lower REE/kg lean tissue (= <26 kcal/kg lean tissue) compared to the able-bodied group.

### Energy Expenditure During Exercise

**FIGURE 2:**
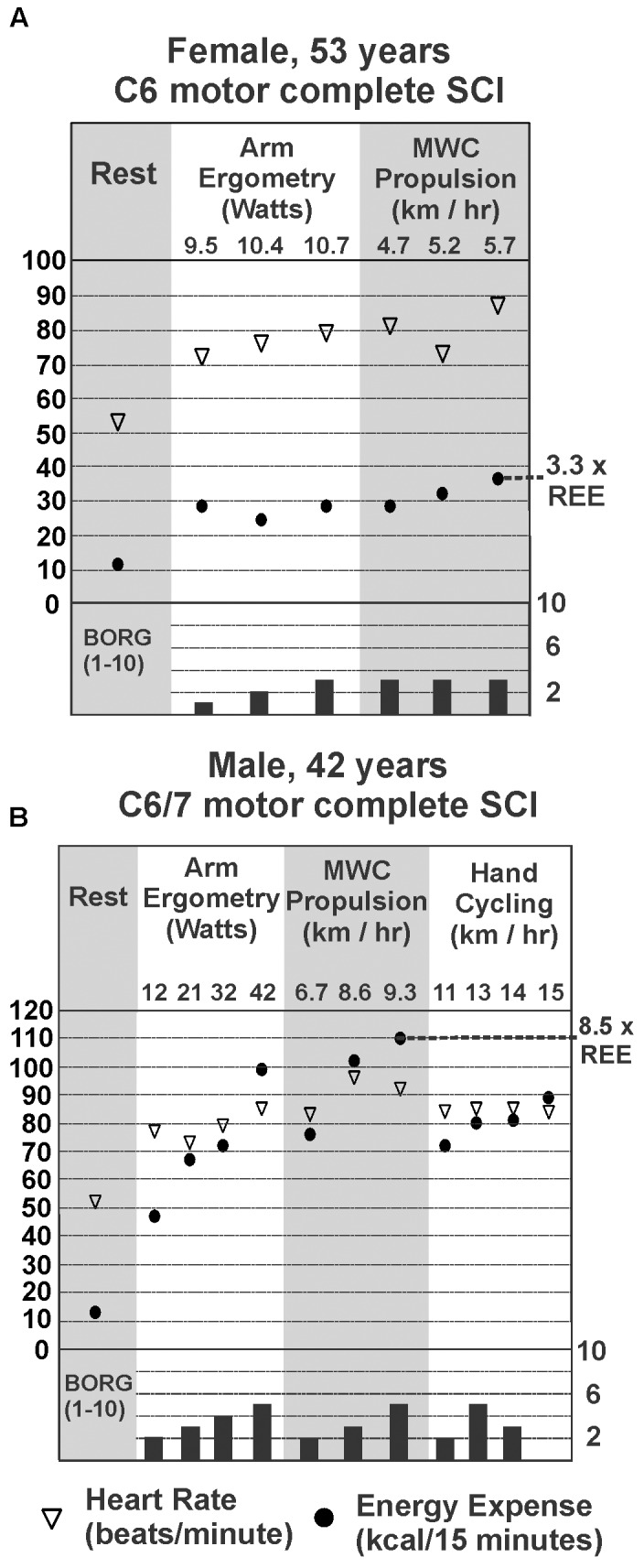
Steady-state energy expenditure (EE) and heart rate (HR) measured at rest (REE; gray panel on left) for a female **(A)** and male **(B)** with motor complete tetraplegia. EE, HR, and Borg rating of perceived exertion for each mode and intensity of exercise measured is shown in panels to the right of resting values. The highest steady state EE measured during exercise for each person is marked by the dotted line and expressed as a multiple of his or her resting EE.

Each participant performed up to four different trials for each exercise mode. Most participants were interested in completing multiple trials and multiple modes of exercise. However, limitations in function prevented those with higher-level injury from completing more than one or two trials. **Figure 2** provides representative examples for a female (**Figure 2A**) and a male (**Figure 2B**) participant with motor complete tetraplegia and include heart rate (empty triangles), energy expenditure (filled circles), and RPE. Note that heart rate and perceived exertion did not consistently increase with increasing power output and energy use, as has been previously reported in tetraplegia (e.g., Valent et al., 2007). **Figure 2** demonstrates the different ranges of exercise intensities achieved by different participants. We observed that those with higher-level tetraplegia showed a smaller range of exercise intensities (e.g., C4/C5 or C6; **Figure 2A**) compared to lower level tetraplegia (e.g., C7, C8 or C6/7; **Figure 2B**), regardless of gender.

**FIGURE 3:**
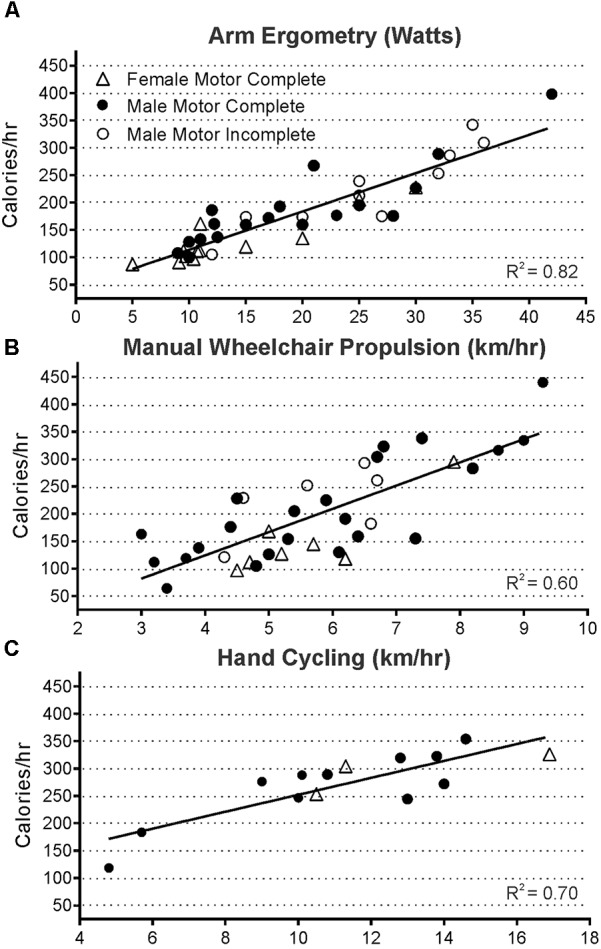
Steady state energy use and power output were well correlated during all modes of exercise measured in 125 exercise trials of arm ergometry (**A**, *p* < 0.0001, *n* = 70 trials), wheeling (**B**, *p* < 0.0001, *n* = 39 trials), and hand cycling (**C**, *p* = 0.0002, *n* = 16 trials). Data from all three groups were pooled since the slopes of each group’s line were not significantly different (male and female motor complete, and male motor incomplete).

**Figure 3** demonstrates that overall, energy use for all participants with tetraplegia ranged from less than 100 kcal/h to just under 450 kcal/h at the intensities they could maintain. Although the absolute range of values was quite low, mean steady state energy use was well correlated with power output for all exercise modes tested [(Evans, 1996); (ergometry *R*^2^ = 0.82, wheeling *R*^2^ = 0.60, and hand-cycling *R*^2^ = 0.70)]. Twenty-two participants with tetraplegia contributed exercise-based energy use data during 70 trials of arm crank ergometry, each at 1 to 4 different power outputs. Steady-state energy use data was also generated during manual wheeling (39 trials with 14 persons each at 2 to 3 different speeds), and hand-bike cycling (16 trials with 5 participants each at 2 to 4 different speeds). Thus, in total, energy use was analyzed during 125 bouts of exercise, in persons with injury levels ranging from C4/C5 to C8. **Figure 3** provides the mean steady-state energy use (kcal/h) for each intensity during arm ergometry (**Figure 3A**), manual wheelchair propulsion (**Figure 3B**), and hand-cycling (**Figure 3C**). Energy use values were averaged for a given power level when more than one person performed at the same power output or speed. Using the median values for each set of tetraplegia-based exercise data, 1 h of arm ergometry at about 15 watts would require ∼150 kcal, and wheeling at just under 6 km/h would require ∼200 kcal. The highest energy use for the median value was observed in hand-cycling such that an hour of cycling at 11 km/h would require ∼275 kcal.

**FIGURE 4:**
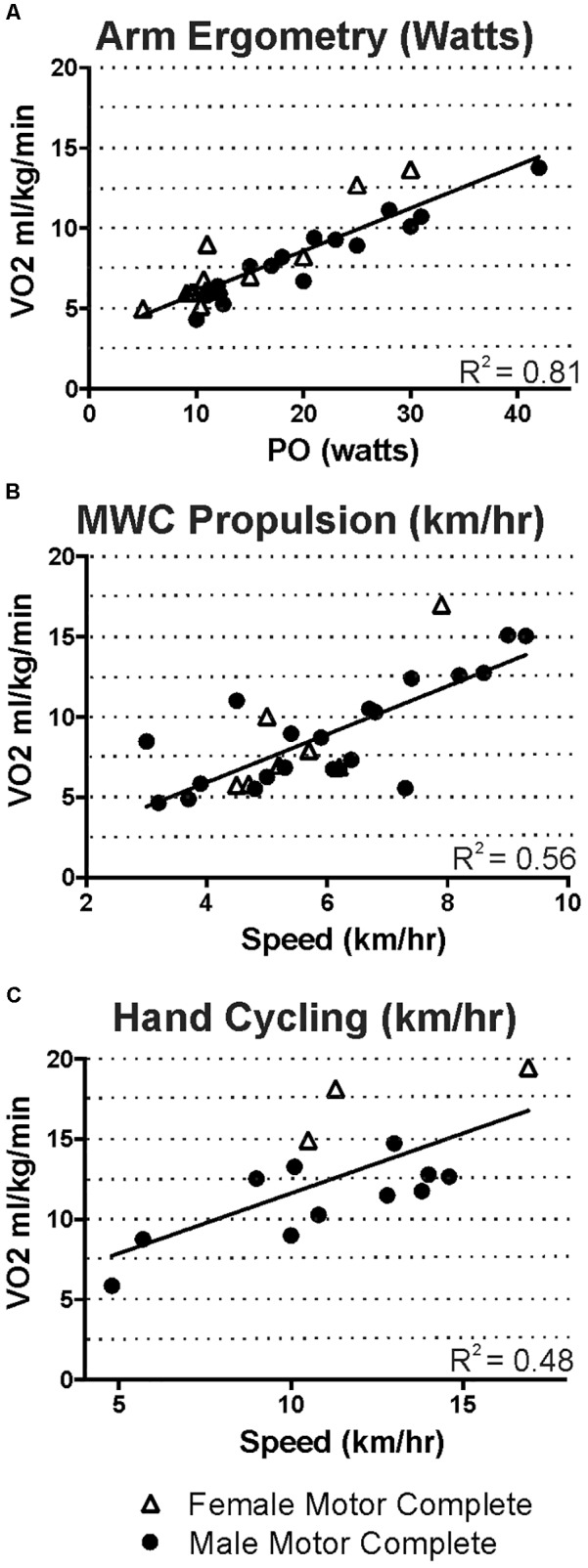
For those with motor-complete tetraplegia, steady state oxygen uptake ranged from 5 to just under 20 ml kg min^-1^ for the intensities that men and women were able to maintain during arm ergometry **(A)**, wheeling **(B),** or hand cycling **(C)**.

**Figure 4** shows the relative oxygen uptake during arm ergometry, wheeling, and hand-cycling for all participants with motor complete tetraplegia. Regression values differ from **Figure 3** because these plots exclude males with motor incomplete tetraplegia and because relative oxygen uptake is increased in participants with less body mass (e.g., compare triangles in **Figures 4C** to **3C**).

### Prevalence of Obesity and Comparison of Different Anthropometric Measures for Assessing Body Fat in Tetraplegia

**FIGURE 5:**
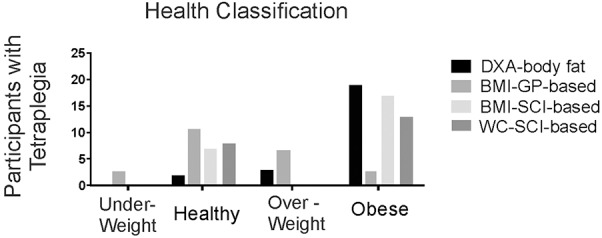
Histogram showing the number of participants with tetraplegia within each body composition classification using either DXA-based total body % fat (DXA-body fat), general population-based body mass index (BMI-GP-based), or SCI-specific classifications based on BMI (BMI-SCI-based) or waist circumference (WC-SCI-based).

Our comparison of anthropometric measures of body fat show a high prevalence of obesity, and that SCI-adjusted BMI appears to be the most useful for determining fat status, in the absence of DXA-based measures. In particular, using gender and age-appropriate DXA-based classification categories (Flegal et al., 2009), only two study participants were classified as healthy whereas 19/24 (79%) were obese, and 3 (12.5%), were overweight (**Figure 5**). As expected, general population-based BMI classification poorly discriminates obesity after SCI (Laughton et al., 2009). In contrast, seventeen would have been classified as obese using the suggested SCI-BMI cut-off of 22 kg m^-2^ (Laughton et al., 2009), and this is closer to the 19/24 that were identified as obese based on DXA-estimates (**Figure 5**). The SCI-specific waist circumference cut-off of 94 cm (Ravensbergen et al., 2014) would have identified 13 as obese in our sample (**Figure 5**). Only 9 to 31% of variance was shared between each of BMI (*R*^2^ = 0.13, *p* = 0.049), waist circumference (*R*^2^ = 0.31, *p* = 0.004) and waist:hip (*R*^2^ = 0.09, *p* = 0.016) and percent body fat in males with motor complete tetraplegia (all *p* > 0.05), but these weak correlations may have been due in part to the high prevalence of obesity in the sample. Thus, in our sample, the SCI-based BMI cut off (Laughton et al., 2009) rather than waist circumference (Ravensbergen et al., 2014) more accurately identified obese individuals.

### Comparison of General Population-Based Estimates of Total Daily Energy Expenditure to Tetraplegia-Specific Estimates of Energy Expenditure

**FIGURE 6:**
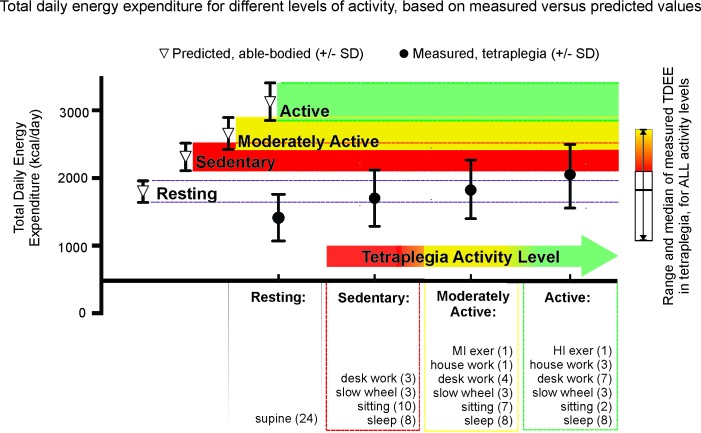
Mean and standard deviation of total daily energy expenditure (TDEE) based on average measured tetraplegia energy consumption demonstrates the low levels of energy expenditure even with very high levels of activity. For each level of activity, the differences were significant between the measured tetraplegia-values and the predicted able-bodied values (*p* = 0.0024 for sedentary, *p* = 0.0003 for moderately active, *p* < 0.0001 for active) but were NS between the measured active and predicted sedentary groups. *t*-Test comparison of total daily energy expenditure at rest was also significantly different (*p* = 0.0031).

We used our measured energy expenditure data as the basis for estimating the size of the energy difference between predicted TDEE and our measured tetraplegia values with increasing levels of physical activity. **Figure 6** shows TDEE (mean ± SD) for a sedentary level of activity in tetraplegia would average 74% of that predicted for a sedentary able-bodied male of the same height and age (mean = 1703 ± 416 vs. 2310 ± 204 kcal for predicted). TDEE increases with increasing activity levels (moderately active mean = 1833 ± 435 vs. 2959 ± 235 kcal for predicted). Increasing activity to include an hour of high intensity activity (3.5 × REE) increases TDEE in tetraplegia to 2027 kcal (active mean = 2027 ± 470 vs. 3128 ± 277 kcal for predicted). The average TDEE for women with motor complete tetraplegia at the sedentary level was 1159 kcal (±303 kcal), and is not shown in **Figure 6**.

**Figure 6** demonstrates that the EE values for sedentary through moderately active levels of activity in tetraplegia fall well below predicted sedentary (red) levels of activity for age and height-matched males from the general population (mean TDEE = 74% and 79% of predicted “sedentary,” respectively). It is only when activity levels are in the “tetraplegia-active” range that average TDEE comes within one standard deviation of the mean TDEE derived for a sedentary level of activity in the able-bodied population (mean TDEE = 88% of predicted). The sidebar presents the median and entire range of TDEE values based on measured energy use in males with motor complete tetraplegia. Therefore, some sedentary men with tetraplegia had TDEE ∼1000 kcal or 43% of predicted sedentary values.

## Discussion

In agreement with previous studies, we show resting energy use in tetraplegia to be significantly lower than predicted compared to the general population, and additionally determined that this decrease was not entirely explained by reductions in lean tissue mass. The present study provides a graphical compilation of absolute steady-state energy use values over a range of modes and known intensities of exercise that reflects the steady-state exercise capabilities of those with motor complete tetraplegia-level SCI. We showed the high prevalence of obesity based on percent body fat and that next to DXA, SCI-adjusted BMI most correctly identified obese individuals in our sample. Finally, we estimated total daily energy needs (TDEE) using our measured values and increasing levels of activity and compared them to TDEE predicted by age, height and gender from the general population to provide a tool for calculating TDEE that reflects realistic levels of activity after tetraplegia. The implications of these findings are discussed further below.

### REE Is on Average Similar to Paraplegia-Level Injury, but There Is a Subgroup With Significantly Lower REE

We report here that the average REE in either males or females with motor-complete tetraplegia is 22% less than predicted or measured in comparable adults from the general population. These numbers are consistent with other studies of males with SCI reported previously (Mollinger et al., 1985; Sedlock and Laventure, 1990; Monroe et al., 1998; Buchholz et al., 2003; Jeon et al., 2003; Nevin et al., 2016). Our finding of a significant difference between those with tetraplegia and the able-bodied reference group after normalizing REE to lean tissue mass is consistent with the findings in paraplegia (Sedlock and Laventure, 1990; Monroe et al., 1998). To our knowledge, this is the first report to include data of lean tissue and REE in women with tetraplegia. In terms of relative oxygen uptake, we measured 2.59 ± 0.60 ml kg min^-1^ in the males with motor complete tetraplegia, which is similar to the two values previously reported [2.54 ± 0.22 (Holmlund et al., 2018) and 2.52 ± 0.5 (Collins et al., 2010)]. Our value of 2.18 ± 0.60 for women with motor complete tetraplegia is lower than the only other study we can compare to [2.60 ± 0.36, (Holmlund et al., 2018)], but may be due to the small sample size for both studies [*n* = 6 in this report and *n* = 7 in Holmlund et al., 2018].

One of the findings from this research report concerns the very low resting values measured in a sub-group of persons with tetraplegia. Specifically, almost half of our sample (9/22) had REE under 1200 cal/day (these nine averaged 39% less than predicted resting values), and after adjusting for lean tissue mass, six of these persons still had REE less than 28 cal/kg lean tissue (**Figure 1C**). The implications of this observation are twofold: first, it is possible for some persons with even high-level SCI (tetraplegia) to demonstrate normal levels of REE in terms of lean tissue mass, and this may explain why some studies show no difference between those with SCI and the general population once fat free mass is considered (Buchholz et al., 2003; Jeon et al., 2003; Yilmaz et al., 2007).

Second, our observation of a sub-group of study participants with reduced REE per kg of lean tissue is also consistent with previous small (*n* = 10 or 4, respectively) studies (Sedlock and Laventure, 1990; Monroe et al., 1998). This suggests the need for further research to confirm these findings, and to identify and ameliorate factors that may predispose a sub-group with SCI to such extremely low levels of resting energy use. Studies in able-bodied adults indicate that 79–83% of the variation in REE could be attributed to variation in the total mass of metabolically active cells, independent of age and sex (Roza and Shizgal, 1984; Bogardus et al., 1986). The three most metabolically active tissues or regions at rest are skeletal muscle (30%), the splanchnic region (25%), and the brain (20%) (Wade and Bishop, 1962). It is possible therefore, given the relative and absolute decrease in skeletal muscle mass and activity after SCI, and the relative increase in oxygen uptake by homeostasis-related organs such as the liver, that non-skeletal metabolically active tissue will make a relatively larger contribution in measured resting energy. Although we examined our dataset for trends in overall percent body fat, percent leg fat, injury duration, age, and absolute lean tissue mass in the legs, the numbers in this study were too small to identify any such factor.

### Steady-State Exercise-Based Energy Expenditure Is Low for All Modes and Intensities of Exercise That Could Be Maintained by Those With Tetraplegia

Energy expenditure (EE) during all modes and intensities of exercise tested ranged from just over 50 kcal h^-1^ to just under 450 kcal h^-1^ (**Figure 3**). These energy expenditure values reflect the range of intensities and exercise modes most likely to be performed by those with tetraplegia-level SCI, and this information could be used for designing tetraplegia-specific exercise strategies. In absolute energy use terms, exercising at the highest recommended SCI-guideline level [5 weekly 44 min sessions, or 220 min; (van der Scheer et al., 2017)] at the median power output measured in our sample would lead to a *weekly* exercise-based EE of 563 kcal performing arm ergometry (15 watts, ∼150 kcal/h), 750 kcal wheeling (∼6 km/h, ∼205 kcal/h) or 1031 kcal hand-cycling (11 km/h, ∼275 kcal/h). For comparison, mean energy use of 250 kcal/h was reported in motor complete men with tetraplegia practicing rugby (Abel et al., 2008). These numbers fall far short of the recommended 2000 kcal weekly goal, and the expenditure would be even less if the 150 min recommendation for the general population were followed (Paffenbarger et al., 1993; Who, 2010; Tremblay et al., 2011).

Our steady state exercise-based energy values in men and women with tetraplegia are presented graphically to allow estimation of EE over the range of intensities and different modes examined (**Figure 3**). Comparisons to previous work are limited due to the scarcity of research in tetraplegia-level SCI. Specifically, Collins et al. (2010), measured energy use at three intensities of arm ergometry and while wheeling at undocumented speeds in males with motor complete tetraplegia (in 3 to 9 people). Our values (**Figure 4**) are similar to those reported by Collins et al. (2010), for arm ergometry (e.g., 7.01 ml kg^-1^ min^-1^ at 16 watts) and their value for wheeling indoors (6.29 ml kg^-1^ min^-1^) would correspond to our value at ∼4 km/h. To our knowledge, the only other study examining exercise-based energy use in 26 males and females with tetraplegia is that of Holmlund et al. (2018), and their reported averages for two arm ergometer intensities (“low” 7.46 ml kg^-1^ min^-1^ at 10 W; or “high” 8.18 ml kg^-1^ min^-1^ at 20 W), indoor wheeling (e.g., 7.46 ml^.^kg^-1.^min^-1^ at 4.0 km/h), and outdoor wheeling (7.9 ml^.^kg^-1.^min^-1^ at ∼5.1 km/h and 9.6 ml^.^kg^-1.^min^-1^ at ∼7.1 km/h) are similar to our values at these speeds (**Figure 4**). Thus, although our and earlier studies involve relatively small numbers of participants, the findings are consistent for similar intensities of activity (Collins et al., 2010; Holmlund et al., 2018).

Our report reflects a wider range of intensities of exercise compared to the previous reports (Collins et al., 2010; Holmlund et al., 2018). The highest relative oxygen uptake reported by Holmlund et al. (2018) was just under 10 ml^.^kg^-1.^min^-1^ (wheeling) and they report exercise could increase EE up to six times in their sample. Our highest steady-state oxygen uptake values during arm ergometry, wheeling, or hand-cycling were approximately 14–15 ml^.^kg^-1.^min^-1^ for our motor complete male sub-group and 17–19 ml^.^kg^-1.^min^-1^ in the female subgroup (**Figure 4**), and these values represented increases over eight times resting in some cases (e.g., **Figure 2B**). These differences may exist because our participants self-selected their exercise intensities. Also, our study sample consisted of people with a wide range of abilities, and these higher values were measured in one current and three former Paralympic-level rugby or track athletes. Although we did not perform peak exercise capacity tests, it is interesting that the highest steady-state values (14–19 ml^.^kg^-1.^min^-1^) observed in some of our study participants were close to the peak oxygen uptake values reported in other studies of elite trained tetraplegia-level athletes, which range from 10.4 to 19.8 (Bhambhani, 2002; Goosey-Tolfrey et al., 2006), suggesting that those with tetraplegia may often function at, or very close to, their peak aerobic capacity. Our highest steady state values are also consistent with peak values reported for both trained and untrained persons with tetraplegia, in which most measured peak VO_2_ values were between 5 and 20 ml^.^kg^-1.^min^-1^ (Eriksson et al., 1988; Totosy de Zepetnek et al., 2016). Some higher values have been reported but it is unknown if the higher peak values were observed in those with motor-incomplete tetraplegia. For comparison, trained male wheelchair racers with paraplegia exhibit peak oxygen uptake values ranging from 34 to 51 ml^.^kg^-1.^min^-1^ (reviewed in Bhambhani, 2002) and maximal power output of paraplegia-level injury can be more than twice that of tetraplegia during arm crank ergometry (85 versus 40 watts; reviewed in Haisma et al., 2006). The steady state energy values reported here are similar to exercise-based values in those with paraplegia for arm ergometry up to 40 watts, but exercise in paraplegia can be maintained at much higher intensity levels, with associated increased energy use (Abel et al., 2008; Collins et al., 2010).

### Estimated Versus Predicted Total Daily Energy Expenditure and the Need for Tetraplegia-Specific Dietary Guidelines

We demonstrate that large differences exist between predicted and estimated TDEE for each hypothetical activity level after motor complete tetraplegia. If a high level of activity is maintained (tetraplegia active category), the difference between it and the predicted sedentary TDEE is reduced to 88% of predicted (a difference of 283 kcal/day). However, this “active” category included spending 1 h daily exercising at the person’s highest steady-state intensity possible (mean = 3.5 × REE) and would require a significant weekly exercise time (420 min). Being “moderately active” involves 1 h of moderate exercise (mean = 2.4 × REE), and would, on average, relate to daily caloric intake at 79% of predicted, able-bodied sedentary values. For those with tetraplegia, long-term commitment to this high level of activity may be difficult to maintain, and may increase risk of overuse injury.

Therefore, on average, for sedentary levels of activity after tetraplegia, maintaining a healthy body composition will require calorie reduction by 26% (mean reduction to ∼74%) of predicted, sedentary, general population based TDEE. For those with substantively lower REE (i.e., the subgroup of 9/22 with REE < 1200 kcal/day) and reduced capacity for exercise, further reductions in energy intake would be required. For example, the TDEE of the lowest 25th percentile of the males with motor complete tetraplegia within the “sedentary” classification was 1387 kcal/day, or 66% of predicted values. Therefore to prevent weight gain, 25% of male participants would need to keep daily caloric intake below 1387 kcal (range = 1083–1387). Further, if weight loss were a goal, creating a calorie deficit would require additional daily caloric reductions (range 783–1087 kcal/day for a 300 kcal deficit). Although women were not included in our comparison, TDEE was estimated, and at the sedentary level of activity, 2/6 were under 1000 kcal. Such low levels of calorie intake would be justifiably concerning to those working in SCI rehabilitation and counseling, and should emphasize the need to carefully examine dietary choices to ensure sufficient intake of important and essential micronutrients.

Malnutrition, as defined by an excess of caloric intake, coupled with micronutrient deficiencies, has been recognized as a serious concern for those with SCI in general (Kressler et al., 2014), and daily food intake logs indicate that many persons with tetraplegia are already low in many essential micronutrients (Groah et al., 2009). This occurs in those already reporting less calorie consumption than recommended by general population guidelines, and with 74% of the sample being either obese or overweight (Groah et al., 2009). Online SCI resources indicate there should be a reduction in body weight (∼10–15% less) after tetraplegia, and that there should be a reduction in daily caloric intake, recommending a decrease to 23 kcal/kg of ideal weight, but that for those requiring less than 1200 cal/day to seek professional guidance (Lagerstrom and Wahman, 2014; Lagerstrom, 2017). Taken together, these findings suggest that the development of specific guidelines for low macronutrient intake (<1200 kcal/day), and that also provide sufficient essential and important micronutrients for long-term health in tetraplegia are needed. Given that REE does not differ greatly between tetra- and paraplegia level SCI, inactive persons with paraplegia who do not engage in significant amounts of physical activity at sufficient intensities to provide for adequate energy expenditure would also benefit from such nutrient guidelines.

### Optimizing Exercise Strategies in Future Exercise Interventions for Those Living With Tetraplegia

Given the low exercise-based energy use findings reported here, it is likely that the current SCI-specific recommendations for exercise (Martin Ginis et al., 2017; van der Scheer et al., 2017) will need to be increased for health benefit in many of those with tetraplegia. This is supported by the observation that 25 min of daily “moderate intensity” exercise reduced body mass index, percent body fat, and insulin resistance in participants with para-, but not tetraplegia, when compared to inactive groups with SCI (Buchholz et al., 2009). These findings also highlight the need to identify modes of exercise that optimize both workload volume and absolute power output while minimizing risk of musculoskeletal injury. For example, attempting to exercise by wheeling a manual chair outdoors may be sub-optimal because this form of exercise requires shoulder mechanics and muscle forces that can increase risk of injury when dealing with steep inclines (Gagnon et al., 2015), and even low intensity wheeling requires substantial forces from rotator cuff muscles (Veeger et al., 2002). Therefore, other exercise modes with higher mechanical efficiency, such as arm ergometry or hand-cycling, may provide a better means for performing longer duration exercise with less risk of injury (Arnet et al., 2013). Incorporating high intensity interval training (“HIIT”) with steady-state exercise may increase the likelihood of health benefits (Nightingale et al., 2017). Other strategies taken from competitive athletes to increase exercise duration may be useful, such as cooling to deal with thermoregulatory sequelae of tetraplegia (Griggs et al., 2014). Finally, the use of electrical stimulation of paralyzed leg muscles for either low force contraction (Woelfel et al., 2017) or higher force activities such as cycling or rowing should be considered as a necessary adjunct to voluntary exercise. The supplement of electrical stimulation-based exercise should increase energy use, decrease overall body fat and perhaps in the case of rowing, even increase cardiovascular fitness in tetraplegia (Taylor et al., 2014; Gibbons et al., 2016; Dolbow and Credeur, 2017; Dolbow et al., 2017). Given that bouts of voluntary exercise in those with tetraplegia are typically limited to a few minutes of continuous activity (Gass et al., 1980), it will be important to incorporate as many strategies as possible to optimize the likelihood of generating a health benefit from exercise.

## Conclusion

Our findings demonstrate the low energy expenditure of people with tetraplegia at rest, and during a wide range of modes and intensities of steady state exercise. These findings, and our comparative analysis of TDEE predicted for the general population, support the need to develop tetraplegia-specific energy balance guidelines. It will be important to design nutrition guidelines that ensure sufficient micronutrients for health, given the low macronutrient intake needed to reduce obesity during the many years typically lived after SCI. Our findings also highlight the need for, and can be used to design, future randomized controlled trials attempting to identify the work volume needed to confer an exercise-related health benefit in individuals living with tetraplegia.

## Author Contributions

KC and BS designed the study. JL provided expertise and participated in pilot testing, developing protocols, and training on data collection. JS performed the data analysis, and contributed to manuscript and figure preparation. KC was responsible for final manuscript preparation. All authors contributed to manuscript revisions, read, and approved the submitted manuscript.

## Conflict of Interest Statement

The authors declare that the research was conducted in the absence of any commercial or financial relationships that could be construed as a potential conflict of interest.

## Footnotes

1 http://www.who.int/features/qa/malnutrition/en/ (accessed July 29, 2018).

2 https://www.cdc.gov/healthyweight/effects/index.html (accessed July 29, 2018).

3 https://health.gov/paguidelines/guidelines/summary.aspx (accessed July 29, 2018).
